# Experimental Analysis of Temperature-Control Curtain Regulating Outflow Temperature in a Thermal-Stratified Reservoir

**DOI:** 10.3390/ijerph19159472

**Published:** 2022-08-02

**Authors:** Chunxi Liu, Jijian Lian, Haijun Wang

**Affiliations:** 1 State Key Laboratory of Hydraulic Engineering Simulation and Safety, Tianjin University, Tianjin 300072, China; liuchunxi@tju.edu.cn (C.L.); bookwhj@163.com (H.W.); 2 Marine Energy and Intelligent Construction Research Institute, Tianjin University of Technology, Tianjin 300382, China

**Keywords:** temperature-control curtain (TCC), outflow temperature, thermal stratification, physical model test, thermal-stratified reservoir

## Abstract

The construction of reservoir dams has changed the environment and natural properties of the river course, and deep-water reservoirs present an obvious phenomenon of thermal stratification. Low-temperature outflow water in spring and summer will have a negative impact on the downstream ecological environment. Therefore, it is necessary to take selective withdrawal measures to regulate low-temperature outflow water. The temperature-control curtain project has the advantages of low cost, convenient construction and wide application. Based on the topographic data, a laboratory test model for regulating outflow temperature by a temperature-control curtain is established. A high-power electric heating system is adopted to form a nonlinear thermal stratification. The accuracy of the test data is verified by the prototype observed water temperature. The main parameters affecting the outflow temperature are investigated, including thermal stratification, flow height above the temperature-control curtain, water level, and discharge flow. The results show the following: firstly, the outflow temperature mainly depends on the thermal stratification, decreases with the increase of water level, and increases with the increase of discharge flow; secondly, the effect of a temperature-control curtain on improving the outflow temperature is directly related to the thermal stratification in different months, and the improvement effect is better in spring and summer; finally, the improvement effect increases with the decrease of flow height above the temperature-control curtain, increases with the increase of water level, and decreases with the increase of discharge flow.

## 1. Introduction

The construction of hydraulic and hydropower projects contributes to the development and utilization of water resources, which has important economic and social benefits [1,2]. However, the existence of reservoirs has changed the natural properties of rivers, and the water temperature in deep-water reservoirs shows an obvious vertical thermal stratification phenomenon [3,4]. In spring and summer, the surface water temperature is higher because of strong solar radiation and high air temperature [5]. The water temperature in the middle and lower layers is relatively low due to the large water depth, slow flow rate, and insufficient heat transfer capacity [6]. The elevation of the water intake in a traditional hydropower station is low, and the thermal stratification phenomenon causes the outflow temperature to be lower than that of natural rivers in spring and summer [7,8,9]. The variation of water temperature may bring negative ecological and environmental problems [10]. Hydraulic and hydropower projects need to meet the economic growth of human society while maintaining the sustainability of ecosystems and biodiversity [11]. Therefore, the treatment and regulation of low-temperature outflow water is an urgent water environment engineering subject [12,13].

Before the dam construction, the water depth of the natural river is shallow and the flow velocity is large, so the water heat exchange is relatively sufficient. The surface and bottom water temperatures of the river are basically the same, and there is no thermal stratification. After the reservoir is built and impounded, due to the large water depth and insufficient heat exchange, there is a temperature difference between the surface and the bottom layer. The water temperature in spring and summer is affected by solar radiation and external temperature. The surface water has a higher temperature and a lower density, while the bottom water has a lower temperature and a higher density. This situation is obviously different from the natural river temperature, resulting in the vertical thermal stratification phenomenon.

According to the thermal stratification distribution, the reservoir water can be vertically divided into three parts: surface temperature layer, thermocline layer and hysteresis layer. The thermal stratification of thermocline changes significantly, and the temperature gradient is large. If the temperature difference between the surface and the hysteresis layer is small, and the temperature gradient in the thermocline is small, it is weak stratification distribution. If the temperature in the surface layer and hysteresis layer is basically unchanged, and the temperature gradient in the thermocline is large, it is a single thermo-cline distribution. If the temperature in the hysteresis layer is basically unchanged, and the temperature gradient of the surface layer and the thermocline is large, it is a double thermocline distribution.

In order to alleviate the negative effects of low-temperature outflow water, various engineering measures have been proposed by domestic and foreign scholars. Typical ones include selective withdrawal facilities [14], aeration facilities for destroying thermal stratification [15], and hydro-ecological regulation facilities [16], among which selective withdrawal facilities are the most widely used. Temperature-control curtains are an effective selective withdrawal facility. Compared with other traditional selective withdrawal facilities, TCCs have an advantage in economic budget [17]. TCCs can be installed in front of the water intake, which will greatly reduce the cost of dismantling the original water intake and rebuilding other types of selective withdrawal facilities [18]. TCCs have the advantages of low construction cost, the construction of a water storage environment, simple structure and wide applicability [19].

**Figure 1:**
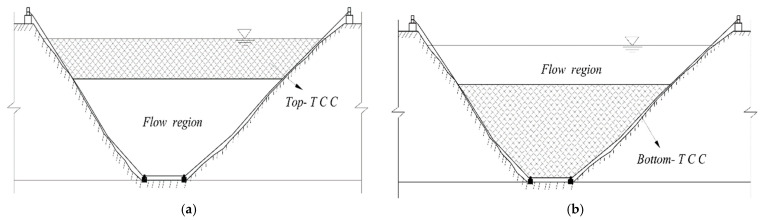
Schematics about different types of TCC: (**a**) Top-TCC; (**b**) Bottom-TCC.

TCCs can be classified into two categories based on their various positions: top-TCC and bottom-TCC (Figure 1). Top-TCC can effectively improve water quality, prevent algae from breeding and reduce outflow temperature. The TCC has been increasingly used to change the temperature of outflow water due to its low cost and ease of construction. A bottom-TCC is made up of a bottom gravity anchorage system, a main cable, a water-proof curtain wall, and a floating bridge system.

Some small reservoirs had only spillways, such as the Lewiston reservoir in the United States [20]. The spillway was equipped with a top-TCC device, which aimed to block the high-temperature water in summer, reduce the outflow temperature and improve the living environment of downstream cold-water fish. Politano et al. conducted a three-dimensional simulation for McNary Dam to study the thermal stratification in front of the dam and the outflow temperature after the application of TCC at the intake [21]. Shammaa found that jets form under TCC in a certain area and there was a strong water exchange cycle, which was verified in the two-layer flow model [22]. Lian Jijian et al. established a three-dimensional thermal stratification simulation model based on flow-3D software, and analyzed the laws and main influencing factors of bottom-TCC to change the outflow temperature [23]. He Wei et al. carried out the mechanism of thermal stratification flow in front of the dam and the traceability analysis of outflow temperature, and obtained the influencing factors and variation rules of outflow temperature [24].

Currently, the main research methods for thermal stratification reservoirs are the empirical formula method [25], the simulation model method [26], and the physical model test method [27]. In early physical model tests, salt and fresh water were used to form density stratification, which was usually divided into two layers along the water depth to simulate water with different temperatures [28]. In addition to the two-layer stratification model, density linear models were gradually developed [29]. Previous studies have focused on stratified flow problems, such as ultimate suction height, water intake layer thickness, critical Froude number, and critical flow, etc. [30]. However, there is a certain gap between the water temperature stratification model and the actual engineering thermal stratification.

**Figure 2:**
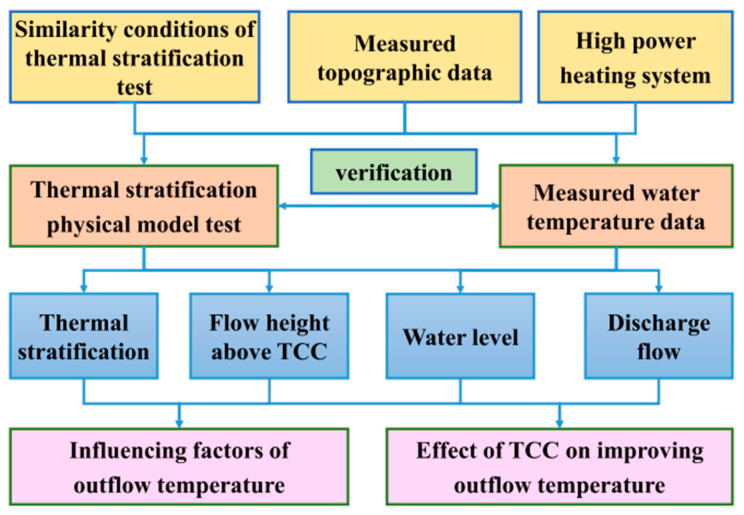
Working program.

In order to achieve water temperature distribution consistent with the prototype, a high-power heating system is used to directly heat the test water in this experiment. The heating effect is observed through a temperature monitoring system. Most of the thermal stratification tests are flume models, and there are few laboratory tests based on actual topography. A laboratory test is established based on actual topographic data in a thermal-stratified reservoir in Southwest China. Compared with the flume model, the nonlinear thermal stratification based on the topographic data in front of the dam is closer to the actual working conditions. The working program is shown in Figure 2. The outflow temperature is affected by various factors, including water level, discharge flow and thermal stratification. This paper investigates the effect of TCC on improving outflow temperature and analyzes the influence of different factors on outflow temperature.

## 2. Materials and Methods

### 2.1. Case Study

**Figure 3:**
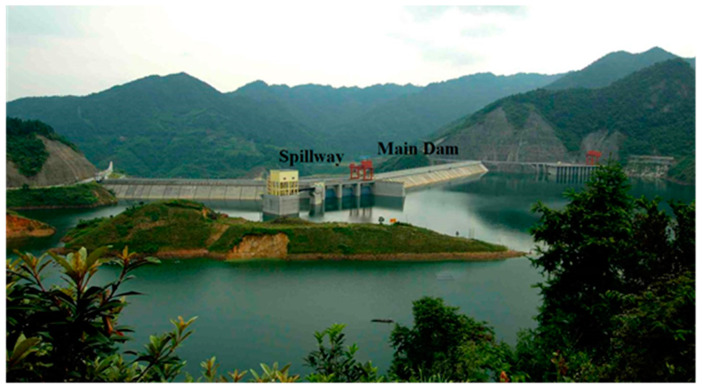
Upstream of hydropower station dam.

**Figure 4:**
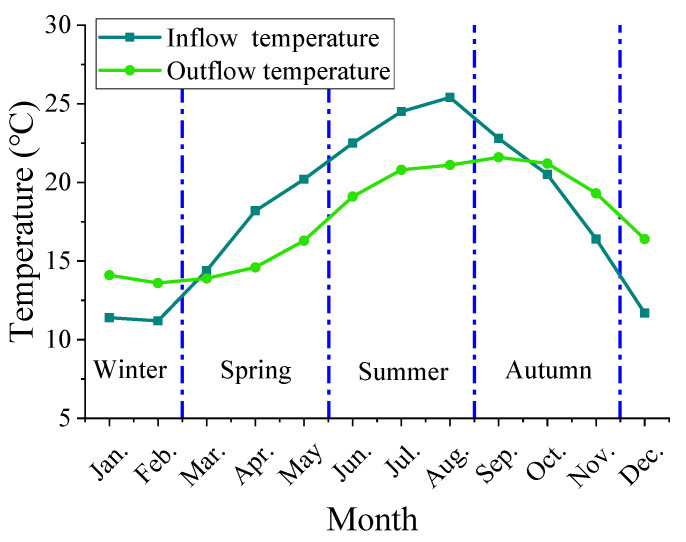
Comparison of monthly average inflow and outflow temperature.

The case study is a deep-water reservoir located in Southwest China which was built and put into operation in 2006. The average water depth in front of the dam is over 130 m. The annual inflow of the reservoir is 7.57 billion m^3^, the normal storage level is 475 m, and the dead water level is 425 m. The hydropower station is equipped with 4 turbine generating units, with a total installed capacity of 1000 MW, a full flow rate of 870 m^3^/s, and a total storage capacity of 4.094 billion m^3^. The water intake elevation of the diversion power generation system is 408 m. The basic parameters of the thermal-stratified reservoir are shown in Table 1. The main dam is a concrete face rockfill dam arranged in a river valley. The height of the dam top is 482.5 m, the lowest height of the toe board is 297.0 m, the maximum height of the dam is 185.5 m, the length of the dam top is 423.3 m, and the width is 10 m. Figure 3 shows the upstream of the hydropower station dam. As the water depth increases, water temperature stratification appears obvious. Due to the low water intake elevation, the stratification of water temperature makes the outflow temperature different from that of the natural incoming flow. Figure 4 shows the comparison of monthly average inflow and outflow temperature. Outflow temperature tends to be lower than natural inflow temperature in spring and summer, and the difference between the outflow and inflow temperature is the largest in August. The inflow and outflow water temperatures were recorded every 10 days.

### 2.2. Test Design

The similarity conditions of thermal stratification laboratory tests are gravity similarity and buoyancy similarity. That is, on the premise of ensuring geometric similarity, gravity Froude number *Fr* and density Froude number *Fd* are equal [31,32]. The gravity Froude number *Fr* ensures the similarity of fluid flow, and the density Froude number *Fd* ensures the similarity of thermal stratification. The temperature difference between the two adjacent layers of the laboratory model is equal to that of the prototype. Then, the conversion relationship between the laboratory model and the prototype is:

$${\bar{T}}_{p}={\bar{T}}_{m}+(T_{Bp}-T_{Bm})$$

where ${\bar{T}}_{p}$ is the prototype outflow temperature (°C); ${\bar{T}}_{m}$ is the laboratory model outflow temperature (°C); $T_{Bp}$ is the basic water temperature at the bottom of the prototype (°C); $T_{Bm}$ is the basic water temperature at the bottom of the laboratory model (°C).

The laboratory test consists of a water tank, a high-power heating system, a water temperature acquisition system, and a flow control device. Considering the laboratory site and test conditions, the geometrical scale is *λ_L_* = 150 (prototype/model). The test design should meet the gravity similarity and buoyancy similarity criteria. The main experimental parameters and geometric scales are shown in Table 2.

**Figure 5:**
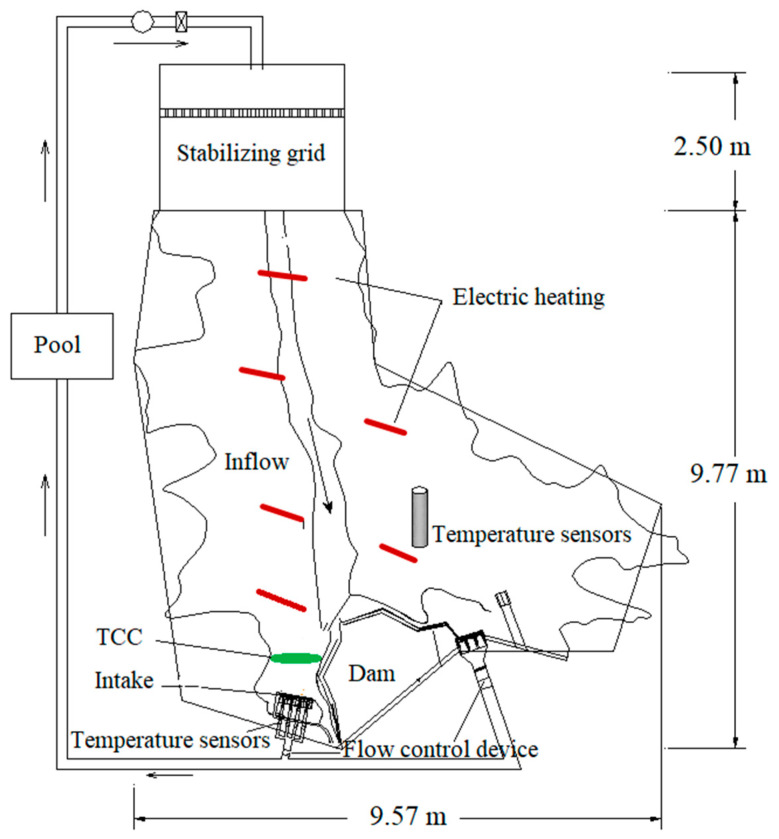
Laboratory test geometry and heating system layout.

**Figure 6:**
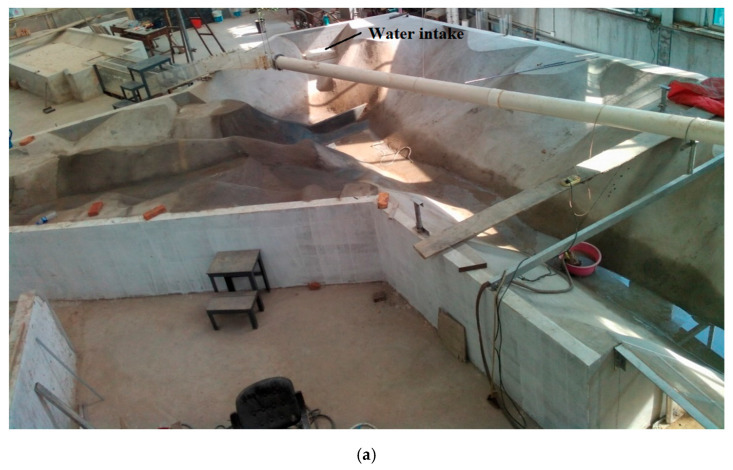

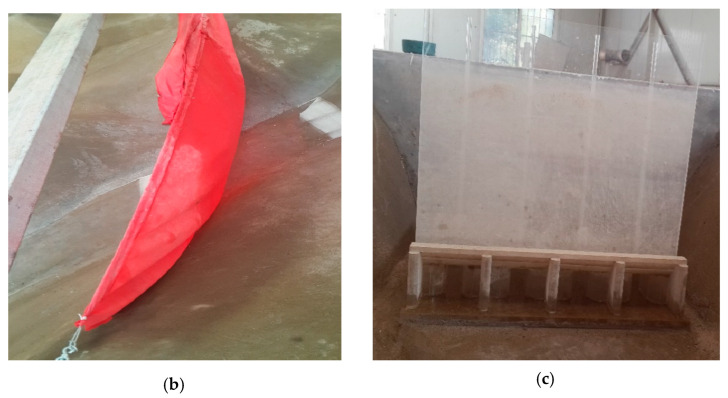
Schematic of TCC experiment: (**a**) Test model terrain; (**b**) TCC; (**c**) Water intake.

The laboratory test model includes a 1.38 km water area in front of the dam, a water intake and a spillway area. The model is 9.77 m long, 9.57 m wide and 1.07 m high. The high-power heating system consists of six fully sealed submersible electric heating tubes, with a heating power of 4–8 kW and a voltage of 380 v. The laboratory test site and the arrangement of the high-power heating system are shown in Figure 5. The water area model in front of the intake was established based on the actual survey geographic data, and the terrain was built by masonry and mortar plastering, as shown in Figure 6.

**Figure 7:**
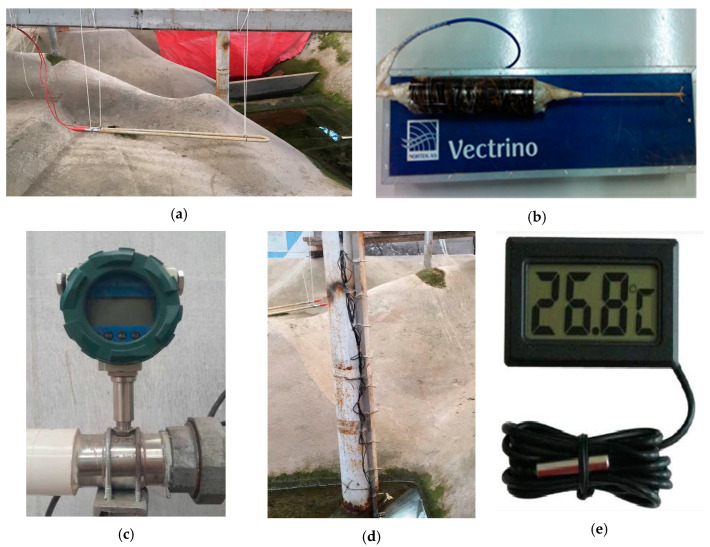
Experimental measuring instruments: (**a**) Electric heating tube, (**b**) ADV, (**c**) Turbine flowmeter, (**d**) Probe arrangement of water temperature sensors, (**e**) Temperature sensor.

The test instruments mainly include high-power electric heating tubes, current meter (ADV), turbine flowmeter, water level measurement system and temperature sensors, as shown in Figure 7. The parameters measured in the laboratory test mainly include water temperature at different depths, outflow temperature, discharge flow, water level, etc. The water level was measured by the water level measurement system and discharge flow is measured by a turbine flowmeter. The flow velocity was measured by a high-precision current meter which has a measurement range of 0–2 m/s, measurement accuracy of ±0.02 cm/s, and maximum sampling frequency of 100 Hz. The measured data of flow velocity were transmitted to the computer acquisition system for real-time recording. The water temperature is measured by digital temperature sensors which have a measurement range of −20–70 °C and measurement accuracy of 0.1 °C. The digital temperature sensors have the advantages of convenient reading, affordable price, corrosion resistance, and so on.

The steps of the test mainly include: (1) pre-test preparation. The circuit equipment is checked. The power of the electric heating tube is large, and the personal safety of the test personnel should be strictly guaranteed during the water temperature heating process. (2) The test model should be watered to the required water level, the water curtain should be installed to the specified position, and the flow height of the TCC should be adjusted. (3) The water temperature heating system is then turned on. During the heating process, the temperature sensor is used to monitor the thermal stratification of the test water until the target thermal stratification is achieved. In order to stabilize the thermal stratification, the heating process takes a long time, about 3–4 h or more. (4) The discharge flow is adjusted according to the test conditions, and the test water level should start to be collect; the thermal stratification data and outflow temperature after the discharge flow indicator is stable. The data are recorded every 30 s for 5 min. (5) Close the discharge flow, adjust the thermal stratification according to the test conditions, and repeat the test.

The thermal stratification of different months is simulated by heating water in this experiment. In order to achieve the target temperature stratification, the electric heating system should meet the requirements of high-power and be fully submersible. According to the temperature monitoring data, the position of the heating tube is adjusted at regular intervals until the thermal stratification in the test matches the actual thermal stratification. The temperature measurement system is composed of many digital temperature sensors. In order to monitor the vertical water temperature, 13 temperature sensors are arranged at equal distances in the test with an interval of 10 cm. Two temperature sensors are arranged at the water intake to measure the outflow temperature. As the test time of each group is about 5 min, the water level is stable and the inlet and outlet flow are similar, and therefore the thermal stratification can be considered to be stable and unchanged during the test.

### 2.3. Test Scenarios

#### 2.3.1. Thermal Stratification

**Figure 8:**
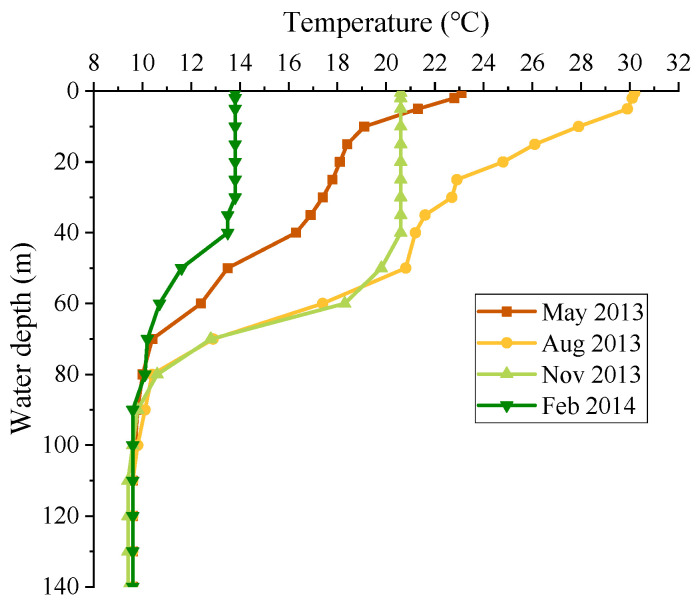
Thermal stratification of representative months.

The thermal-stratified reservoir is located in the area where the temperature is distinct in four seasons. The distribution difference of thermal stratification in each season is large. In order to be rigorous and to save resources, the representative months of each quarter are selected for analysis, and are February, May, August and November, respectively. The thermal stratification distribution of representative months is shown in Figure 8.

As can be seen from Figure 8, the temperature difference between the surface and bottom in February is about 5 °C, and the thermal stratification intensity is weak, belonging to weak stratification. In May, the temperature difference between the surface and bottom is about 14 °C, and the temperature gradient of the surface and thermocline is large, belonging to double thermocline distribution. In August, there is strong solar radiation and high temperature, and the temperature difference is about 21 °C, which belongs to double thermocline distribution. In November, the surface water temperature is about 21 °C, and the temperature difference is about 12 °C. The surface temperature is basically unchanged, while the thermocline temperature changes in a large gradient, which belongs to single thermocline distribution. The thermal stratification in other months is similar to the above four typical months.

#### 2.3.2. Flow Height above TCC

To improve the outflow temperature, the test TCC is arranged at the bottom to block low-temperature water. The TCC is impermeable and its base is consistent with the topography. The TCC is arranged 250 m in front of the intake, which is converted into the prototype size. Different flow heights above TCC will change the outflow temperature. In laboratory tests, three different flow heights of 20 m, 30 m and 40 m are selected to study the influence of flow heights on outflow temperature.

#### 2.3.3. Water Level

The average operating water level of the thermal-stratified reservoir is 465 m. The water level in the dry season is about 445 m, and the water level in the wet season is about 475 m. Four typical water levels of 445 m, 455 m, 465 m and 475 m are selected to study the effects of different water levels on outflow temperature.

#### 2.3.4. Discharge Flow

In addition to the spillway and other discharge facilities during the flood period, the outlet is used for discharge most of the year. The full discharge of the outlet is 870 m^3^/s, and the average inflow in spring and summer is about 500 m^3^/s. The discharge flow in the test is set at 125 m^3^/s, 250 m^3^/s, 500 m^3^/s and 870 m^3^/s respectively.

In laboratory tests, four influencing factors including thermal stratification, flow height above TCC, water level and discharge flow are considered. Spring is the breeding period for fish and the planting season for fields. The rise in water temperature can improve fish spawning reproduction and increase the growth rate of crops. Therefore, the outflow temperature in spring has a great impact on the downstream river environment. The thermal stratification in May, flow height of 30 m, water level of 465 m and discharge flow of 500 m^3^/s are taken as the basic working conditions in the laboratory tests. The influence of thermal stratification, flow height, water level and discharge flow on the outflow temperature are investigated. Simultaneously, no TCC is employed as a comparative condition. The laboratory test scenarios are shown in Table 3.

## 3. Results

### 3.1. Model Verification

**Figure 9:**
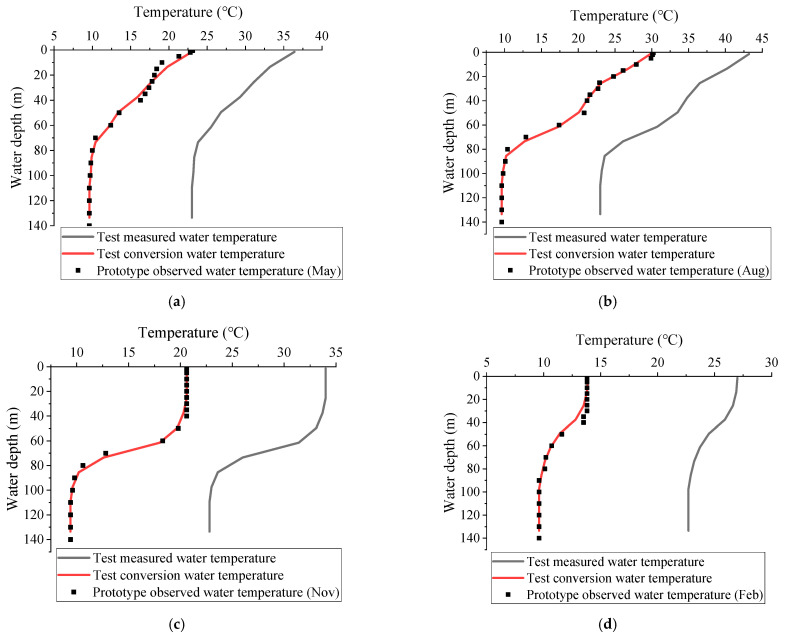
Comparison of measured water temperature, conversion water temperature and prototype observed water temperature: (**a**) May; (**b**) August; (**c**) November; (**d**) Next February.

The key to the reliability of laboratory test results is to simulate the thermal stratification of the prototype. The submersible electric heating tubes are used to directly heat the experimental water in the laboratory test, and the water temperature is monitored until the target thermal stratification is achieved. According to the similar conditions of the thermal stratification test described in Section 2.2, the test thermal stratification is converted to the prototype water temperature. The distribution of thermal stratification in May, August, November and February are taken as typical working conditions. The geometrical scale of the test is *λ_L_* = 150 (prototype/model). The data obtained from the test are transformed into the corresponding parameters of the prototype. This is of reference significance for practical engineering, which is also the purpose of the test. So the test results are based on the test converted data. Table 4 shows the comparison of outflow temperature between test measurement (after conversion) and prototype observation. The comparison of measured water temperature, converted water temperature and prototype water temperature is shown in Figure 9.

As can be seen from Figure 9, the test water temperature after conversion is basically consistent with that of the prototype. The nonlinear thermal stratification distribution can be formed by directly heating water with high-power electric tubes. The thermal stratification of the prototype reservoir is well simulated in laboratory tests, and the two are in good agreement. The relative error of outflow temperature between the test measurement (after conversion) and the prototype data in typical months is small (Table 4). The minimum difference in outflow temperature occurs in February with a difference of 0.1 °C. August has the greatest difference in outflow temperature, with a difference of 0.4 °C. The results of the laboratory test (after conversion) agree well with the prototype observations, and the results of the thermal stratification test are accurate and reliable.

### 3.2. Thermal Stratification

In order to study the influence of thermal stratification on outflow temperature, four thermal stratifications are considered, that is, May, August, November and next February. Other parameters are kept constant and are set as follows: a water level of 465 m, discharge flow of 500 m^3^/s, and flow height above curtain of 30 m. In addition, there are no TCCs designed in the comparison scenarios. The thermal stratification test scenarios and test results are shown in Table 5.

**Figure 10:**
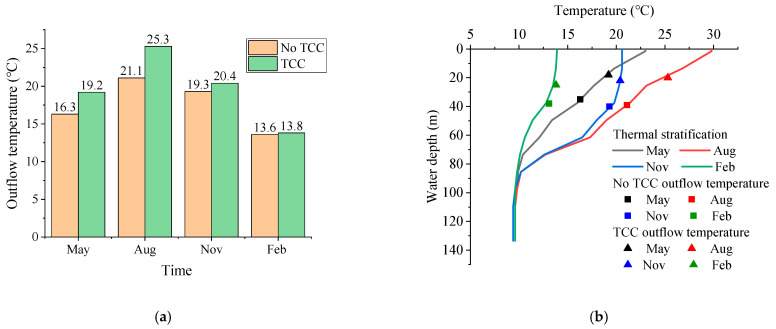
Comparison of outflow temperature: (**a**) before and after TCC implementation; (**b**) different thermal stratification.

Figure 10 shows the comparison of outflow temperature before and after TCC implementation and outflow temperature of different thermal stratification. Compared with no TCCs scenarios, the outflow temperature after TCC implementation in May, August, November and next February increased by 2.9 °C, 4.2 °C, 1.1 °C and 0.2 °C, respectively. In May and August, the water temperature difference between the surface and the bottom layer was substantial, the thermocline was prominent, and the surface temperature changed greatly. After the implementation of TCC, the outflow temperature increased substantially, and the effect of improving outflow temperature was improved.

In November, the temperature difference between the surface and bottom layer was about 12 °C, and the thermocline fluctuated a lot, although the surface water temperature remained rather constant. The TCC had little effect on improving the outflow temperature. The temperature difference between the surface and bottom in February was just about 5 °C. The temperature stratification was weak, and the TCC had only a minor impact on improving the water temperature. The test results demonstrate that the outflow temperature is mostly influenced by thermal stratification. For thermal stratification with large temperature differences (e.g., May and August), the TCC has a good effect on improving the outflow temperature. When the surface water temperature is essentially the same (e.g., November), the TCC can improve the outflow temperature, but only modestly. The TCC has little effect on the outflow temperature when the thermocline gradient changes little (e.g., February). The laboratory test results show that the effect of TCC on improving low-temperature water is greatly affected by thermal stratification. TCCs may significantly improve the outflow temperature in spring and summer, which is an effective selective withdrawal facility.

### 3.3. Flow Height above TCC

To investigate the effect of flow height above TCC on outflow temperature, 20 m, 30 m and 40 m heights are chosen as typical working conditions. In the laboratory test, the thermal stratification is May, the water level is 465 m, and the discharge flow is 500 m^3^/s. And there are no TCCs designed in the comparison scenarios. The flow height test scenarios and test results are shown in Table 6.

**Figure 11:**
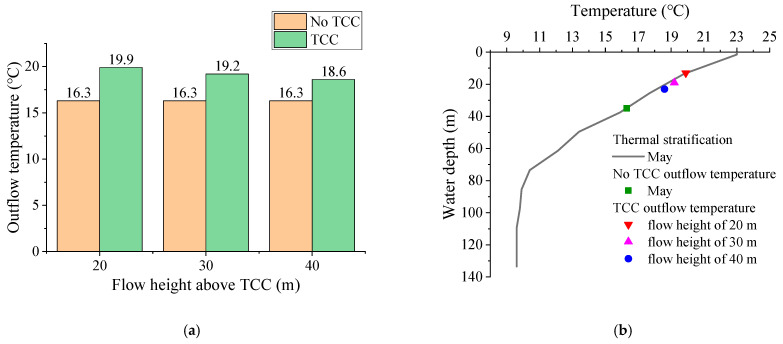
Comparison of outflow temperature: (**a**) before and after TCC implementation; (**b**) different flow height.

Figure 11 shows the comparison of outflow temperature before and after TCC implementation and outflow temperature at different flow heights. The outflow temperature was 16.3 °C without TCC. After the TCC was implemented, the outflow temperatures at the flow height of 20 m, 30 m and 40 m were 19.9 °C, 19.2 °C and 18.6 °C, respectively. The outflow temperature was the highest at 20 m flow height and the lowest at 40 m flow height. Compared with the scenario without TCC, the outflow temperature at the flow height of 20 m, 30 m and 40 m increased by 3.6 °C, 2.9 °C and 2.3 °C, respectively. The results show that for the thermal stratification in May, the construction of TCC can effectively improve the outflow temperature. The lower the flow height, the higher the outflow temperature, and the greater the effect on improving low-temperature water.

### 3.4. Water Level

The water level of the reservoir is dynamic and varies in different seasons. Water levels of 445 m, 455 m, 465 m and 475 m are selected to analyze the effect on outflow temperature. Other impact parameters are kept constant with thermal stratification in May, flow height of 30 m, and discharge flow of 500 m^3^/s. No TCCs are designed in the comparison scenarios. The water level test scenarios and test results are shown in Table 7.

**Figure 12:**
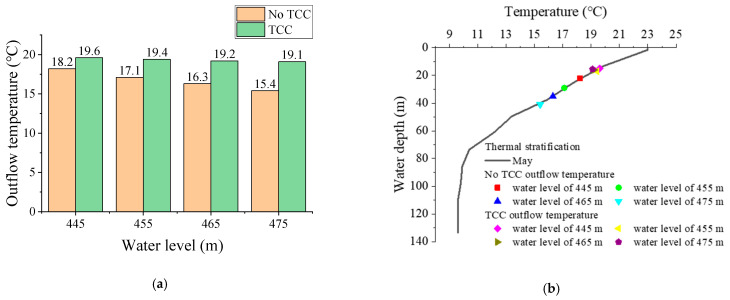
Comparison of outflow temperature: (**a**) before and after TCC implementation; (**b**) different water level.

Figure 12 shows the comparison of outflow temperature before and after TCC implementation and outflow temperature at different water levels. When the water level rose from 445 m to 475 m without TCC, the outflow temperature decreased by 2.8 °C. When the thermal stratification, flow height and discharge flow remain unchanged, the higher the water level, the lower the outflow temperature. After the TCC was implemented, the outflow temperature at the water level of 445 m, 455 m, 465 m and 475 m increased by 1.4 °C, 2.3 °C, 2.9 °C and 3.7 °C, respectively. Compared with the scenarios without TCCs, the higher the water level, the better the effect of TCC on improving water temperature. The outflow temperature difference between the four different water levels with TCC did not exceed 0.5 °C, and the change of water level had little influence on the outflow temperature. This is because when TCC exists, the overflow of high-temperature water at different water levels is basically unchanged. The laboratory test results show that the higher the water level, the lower the outflow temperature in the absence of TCC. However, for the effect of TCC on improving low-temperature water, the higher the water level, the better the effect. After the implementation of TCC, the water level has little effect on the outflow temperature.

### 3.5. Discharge Flow

Discharge flow is one of the important parameters affecting the flow field. To study the effect of discharge flow on outflow temperature, four discharge flows of 125 m^3^/s, 250 m^3^/s, 500 m^3^/s and 870 m^3^/s are considered. Other impact parameters remain unchanged and are set as follows: thermal stratification in May, flow height of 30 m, and water level of 465 m. No TCCs are designed in the comparison scenarios. The discharge flow test scenarios and test results are shown in Table 8.

**Figure 13:**
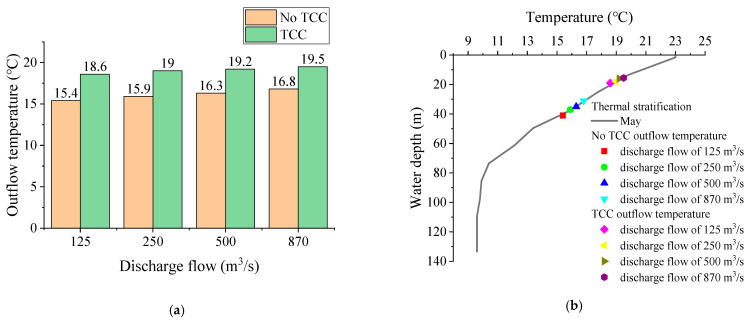
Comparison of outflow temperature: (**a**) before and after TCC implementation; (**b**) different discharge flow.

Figure 13 shows the comparison of outflow temperature before and after TCC implementation and outflow temperature at different discharge flows. When the discharge flow was 125 m^3^/s, 250 m^3^/s, 500 m^3^/s and 870 m^3^/s, the outflow temperatures without TCC were 15.4 °C, 15.9 °C, 16.3 °C and 16.8 °C, respectively. The outflow temperature increases with the increase of discharge flow. After the TCC was implemented, the corresponding outflow temperatures were 18.6 °C, 19.0 °C, 19.2 °C and 19.5 °C, respectively. Compared with the scenarios without TCCs, the outflow temperatures increased by 3.2 °C, 3.1 °C, 2.9 °C and 2.7 °C, respectively. The smaller the discharge flow, the higher the outflow temperature raised by TCCs. The laboratory test results show that the outflow temperature is positively correlated with the discharge flow, while the effect of TCC on improving low-temperature water is negatively correlated with the discharge flow.

## 4. Discussion

Due to the thermal stratification of deep-water reservoirs and the low intake elevation of hydropower stations, the outflow temperature in spring and summer is lower than the inflow temperature. This may have an important impact on farmland irrigation, fish reproduction and water ecological balance. Managers try to take engineering measures to change the flow pattern and reduce the outflow of low-temperature water so as to ensure the normal water environment and water ecology of the downstream river. The TCC project is a competitive new type of selective withdrawal facility.

In this paper, a laboratory test model is designed using actual topographic data in front of the dam of the Southwest Reservoir. The high-power heating system heats the test water directly, producing a temperature distribution that is nearly identical to the prototype nonlinear thermal stratification. Shammaa et al. conducted a flume stratification experiment for TCC using a two-layer flow of fresh and saltwater [22]. Compared with Shammaa’s salinity stratification test, the nonlinear thermal stratification in this test is more consistent with the actual situation. He et al. developed and verified a 3-D thermal tracer model based on Flow-3D and investigated the source of discharged water [24]. The experimental results in this paper are consistent with He’s numerical simulation results.

TCCs have a promising future in the fields of hydropower and coastal engineering. They can improve downstream water quality in addition to managing the temperature of stratified reservoirs. A curtain placed in the upper regions of a river can effectively limit high-nutrient flow into downstream areas, reducing eutrophication and algal bloom [12]. Curtains can also be used to distinguish water of different densities, such as fresh water and seawater, allowing for the construction of marine reservoirs to meet the demand for freshwater in offshore engineering. Flexible materials will become more widespread in hydraulic structures as material science and technology develop.

At present, TCC is still in the design stage and has not been applied in engineering practice. There is a preliminary plan for the construction process of TCC. TCC construction in a full-scale reservoir needs to comprehensively consider the engineering geological environment, construction conditions, TCC structural characteristics, and other factors. The overall construction sequence of TCC is as follows: the part above the water surface is constructed and then the underwater part is constructed. First, the cable towers on both banks are constructed; secondly, the surface pontoon system is arranged, and then the gravity anchor and ground anchor are installed. Finally, the TCC is sunk. The general steps are as follows: (1) construction preparation; (2) construction of cable towers on both banks; (3) positioning and placement of pontoon system; (4) set up water operation platform; (5) install gravity anchors and ground anchors; (6) TCC sinking; (7) commissioning and acceptance project.

## 5. Conclusions

This paper introduces the similarity conditions of the thermal stratification test on the premise of geometric similarity, the gravity Froude number *Fr,* and the density Froude number *Fd* of the model and the prototype are equal. According to similar conditions, actual topographic data and a high-power heating system, a physical test model of a deep-water reservoir is established. The effects of thermal stratification in typical months, flow height above TCC, water level and discharge flow on outflow temperature are analyzed. The main conclusions are as follows:

(1) The thermal stratification and outflow temperature in the test are verified through the prototype-observed water temperature data. The verification results show that the laboratory test can simulate the nonlinear thermal stratification of the actual reservoir, and the test results are accurate and reliable.
(2) The outflow temperature mainly depends on the thermal stratification, decreases with the increase of water level and increases with the increase of discharge flow in the thermal-stratified reservoir.
(3) The effect of TCC on improving the outflow temperature is directly related to the thermal stratification in different months and the improvement effect is greater in spring and summer.
(4) The improvement effect of TCC increases with the decrease of flow height above TCC, increases with the increase of water level, and decreases with the increase of discharge flow.

An experimental study on regulating outflow temperature by TCCs is investigated, but the measured and observed results of the experiment have some limitations. Subsequently, a numerical simulation model should be established and compared with the test results to verify the flow field, temperature field and other information in detail. There are few studies on the law of TCC load, and the interaction mechanism between TCC load and flow is lacking. In the future, the load characteristics of TCC under different working conditions should be studied, which is of reference significance to the research and development of TCC design.

## Figures and Tables

**Table 1:** Basic parameters of the deep water reservoir.

Parameter	Value
Annual runoff	7.57 billion m^3^
Normal water level	475 m
Dead water level	425 m
Maximum discharge flow	870 m^3^/s
Total storage capacity	4.9 billion m^3^
Intake floor elevation	408 m

**Table 2:** Main experimental parameters and geometric scales.

Similarity Criterion	Physical Quantity	Scale Relation	Scale
Gravity similarity criteria	Length	*λ_L_*	150
	Flow	*λ_Q_* = *λ_L_*^2.5^	275,567.6
	Force	*λ_F_* = *λ_L_*^3^	3,375,000
	Time	*λ_t_* = *λ_L_*^0.5^	12.2
Buoyancy similarity criteria	Temperature	*T_H_* − *T_BH_* = *T_M_* − *T_BM_*	-

**Table 3:** The laboratory test scenarios.

NO.	Description	Thermal Stratification	Flow Height (m)	Water Level (m)	Discharge Flow (m^3^/s)
A1	Basic scenario	May 2013	30	465	500
B2	Different thermal stratification	August 2013	30	465	500
B3		November 2013	30	465	500
B4		February 2014	30	465	500
C5	Different flow height	May 2013	20	465	500
C6		May 2013	40	465	500
D7	Different water level	May 2013	30	445	500
D8		May 2013	30	455	500
D9		May 2013	30	475	500
E10	Different discharge flow	May 2013	30	465	125
E11		May 2013	30	465	250
E12		May 2013	30	465	870
F	No TCC	Same as the comparison scenario	-	Same as the comparison scenario	

**Table 4:** Comparison of outflow temperature between test measurement (after conversion) and prototype observation.

Thermal Stratification	Water Level (m)	Discharge Flow (m^3^/s)	Test Outflow Temperature (°C)	Prototype Outflow Temperature (°C)	ΔT (°C)
May 2013	465	500	16.3	16.1	0.2
August 2013	465	500	21.1	20.7	0.4
November 2013	465	500	19.3	19.0	0.3
February 2014	465	500	13.6	13.5	0.1

Note: the water level, discharge flow and test water temperature in the table are all prototype values after conversion.

**Table 5:** Different thermal stratification test scenarios and results.

NO.	Description	Thermal Stratification	Flow Height (m)	Water Level (m)	Discharge Flow (m^3^/s)	Outflow Temperature (°C)
B2	Different thermal stratification	May 2013	30	465	500	19.2 (+2.9)
A1		August 2013	30	465	500	25.3 (+4.2)
B3		November 2013	30	465	500	20.4 (+1.1)
B4		February 2014	30	465	500	13.8 (+0.2)
F13	No TCCs	May 2013	-	465	500	16.3
F14		August 2013	-	465	500	21.1
F15		November 2013	-	465	500	19.3
F16		February 2014	-	465	500	13.6

**Table 6:** Different flow height test scenarios and results.

NO.	Description	Thermal Stratification	Flow Height (m)	Water Level (m)	Discharge Flow (m^3^/s)	Outflow Temperature (°C)
C5	Different flow height	May 2013	20	465	500	19.9 (+3.6)
A1		May 2013	30	465	500	19.2 (+2.9)
C6		May 2013	40	465	500	18.6 (+2.3)
F14	No TCCs	May 2013	-	465	500	16.3

**Table 7:** Different water level test scenarios and results.

NO.	Description	Thermal Stratification	Flow Height (m)	Water Level (m)	Discharge Flow (m^3^/s)	Outflow Temperature (°C)
D7	Different water level	May 2013	30	445	500	19.6 (+1.4)
D8		May 2013	30	455	500	19.4 (+2.3)
A1		May 2013	30	465	500	19.2 (+2.9)
D9		May 2013	30	475	500	19.1 (+3.7)
F17	No TCCs	May 2013	-	445	500	18.2
F18		May 2013	-	455	500	17.1
F14		May 2013	-	465	500	16.3
F19		May 2013	-	475	500	15.4

**Table 8:** Different discharge flow test scenarios and results.

NO.	Description	Thermal Stratification	Flow Height (m)	Water Level (m)	Discharge Flow (m^3^/s)	Outflow Temperature (°C)
E10	Different discharge flow	May 2013	30	465	125	18.6 (+3.2)
E11		May 2013	30	465	250	19.0 (+3.1)
A1		May 2013	30	465	500	19.2 (+2.9)
E12		May 2013	30	465	870	19.5 (+2.7)
F20	No TCCs	May 2013	-	465	125	15.4
F21		May 2013	-	465	250	15.9
F14		May 2013	-	465	500	16.3
F22		May 2013	-	465	870	16.8

## Data Availability

Data are contained within the article.
